# Revitalizing Recovery: Unveiling the Transformative Impact of Physiotherapy in Rehabilitating a Neglected Case of Shoulder Hemiarthroplasty

**DOI:** 10.7759/cureus.53488

**Published:** 2024-02-03

**Authors:** Om C Wadhokar, Minal Dahiwadkar, Sakshi Rawal, Reetkaur Arora, Aakanksha Choudhari, Aishwarya Mali, Chaitanya A Kulkarni, Tushar J Palekar, Mayur Wanjari

**Affiliations:** 1 Public Health, Jawaharlal Nehru Medical College, Datta Meghe Institute of Higher Education and Research, Wardha, IND; 2 Musculoskeletal Physiotherapy, Dr. D. Y. Patil College of Physiotherapy, Pune, IND; 3 Musculoskeletal Sciences, Dr. D. Y. Patil College of Physiotherapy, Pune, IND; 4 Community Based Rehabilitation, Dr. D. Y. Patil College of Physiotherapy, Pune, IND; 5 Community Health Physiotherapy, Ravi Nair Physiotherapy College, Datta Meghe Institute of Higher Education and Research, Wardha, IND; 6 Research and Development, Jawaharlal Nehru Medical College, Datta Meghe Institute of Higher Education and Research, Wardha, IND

**Keywords:** post-operative management, case report, rehabilitation, physiotherapy, shoulder arthroplasty

## Abstract

The shoulder joint has a complex anatomy and biomechanics. It is a ball and socket joint made by the articulation surface of the humeral head (ball) and glenoidal fossa (socket) of the scapula. Shoulder arthroplasty is done when parts of the shoulder joint are severely affected and damaged beyond repair. The damaged parts are replaced with artificial parts. Prosthetic implants are typically made of metal or plastic material. Implants come in various sizes and shapes. There are three types of surgical arthroplasty: total shoulder arthroplasty, partial shoulder arthroplasty, and reverse arthroplasty. Indications of shoulder arthroplasty may include osteoarthritis, fractures, rotator cuff injuries, osteonecrosis, and rheumatoid arthritis. This case study aims to provide a case of anteroinferior dislocation of the left shoulder with humeral head comminuted fracture confirmed by an investigation like radiograph and CT scan operatively managed by left shoulder hemiarthroplasty. In this case study, a 58-year-old male cannot lift his arm and perform actions of the shoulder joint independently after the operative procedure, thus reducing the functional status and quality of life. After the left shoulder hemiarthroplasty repair post due to inadequate rehabilitation, there was a failure in achieving the ranges and gaining back the strength of the muscles. The patient has a combined plan of action, which consists of pharmacological interventions along with physiotherapy rehabilitation. The physiotherapy protocol consists of goals like using electrical muscle stimulation, activation exercises of muscles, strengthening protocol, stretches, and counselling. By the end of the physiotherapy treatment, the patient showed significant progress in re-establishing the ranges and enhanced muscle strength, which resulted in a positive self-boost along with improved functional independence quotient, thereby increasing quality of life.

## Introduction

Shoulder hemiarthroplasty is a surgical procedure that replaces the shoulder joint with artificial materials like metal or plastic to restore function. Some types of shoulder arthroplasty are unconstrained total hemiarthroplasty, semi-constrained inversed shoulder prostheses, and humeral hemiarthroplasty [1]. These surgeries have exhibited <91% success, but about 14% of risks are noted. Some complications are glenoid or humeral head loosening, infection, instability, rotator cuff tear, and ectopic ossification. Revision surgeries are done in cases of complications [2]. Complications of shoulder hemiarthroplasty, according to a study, are pain from glenoid erosion, stiffness, avascular necrosis (AVN), and subscapularis tear. Total shoulder arthroplasty results in glenoid loosening, and shoulder hemiarthroplasty leads to glenoid erosion and stiffness in the long term [3]. After the surgery, range of motion, muscle activity, and proprioception are lost and have to be regained again. Physiotherapy plays a vital role in restoring these functions. For shoulder hemiarthroplasty, restoring the rotator cuff muscle strengthening and function is of utmost importance [4]. It also plays a vital role in shoulder arthroplasty patients, reinstating their shoulder range of motion, mobility, and activities of daily living [5].

## Case presentation

**Figure 1:**
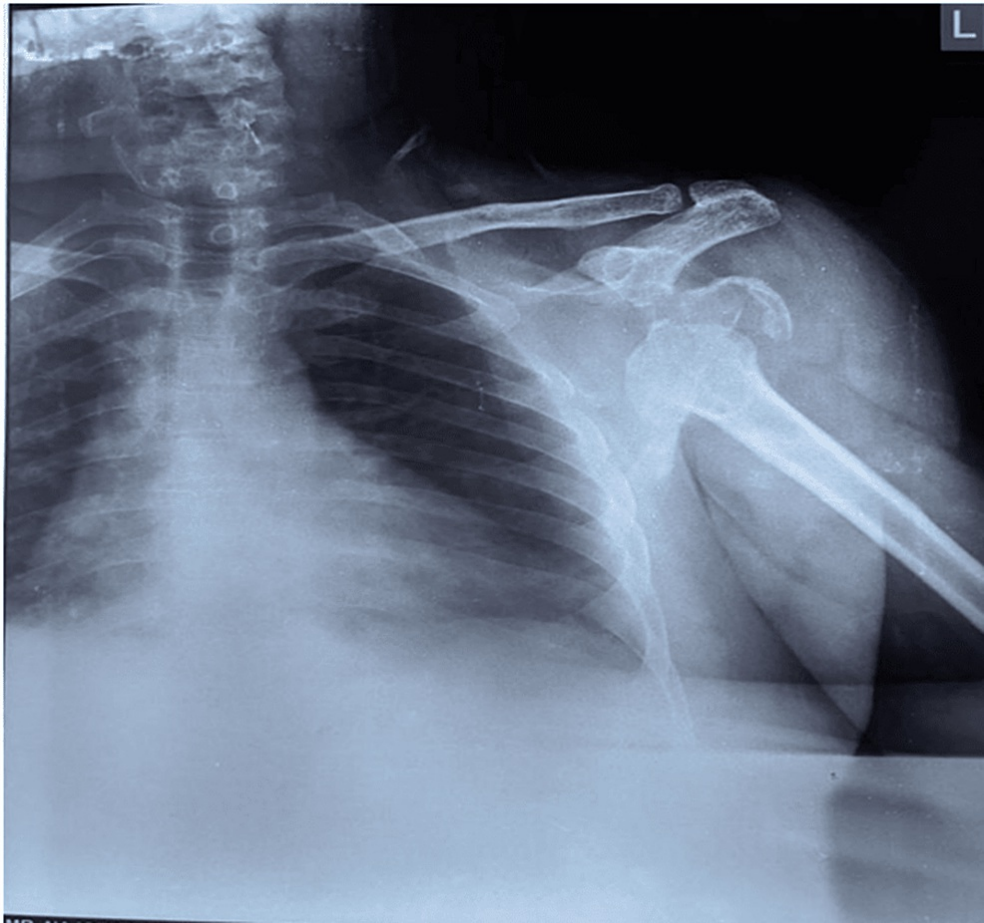
X-ray showing dislocation and fracture of the left shoulder joint.

**Figure 2:**
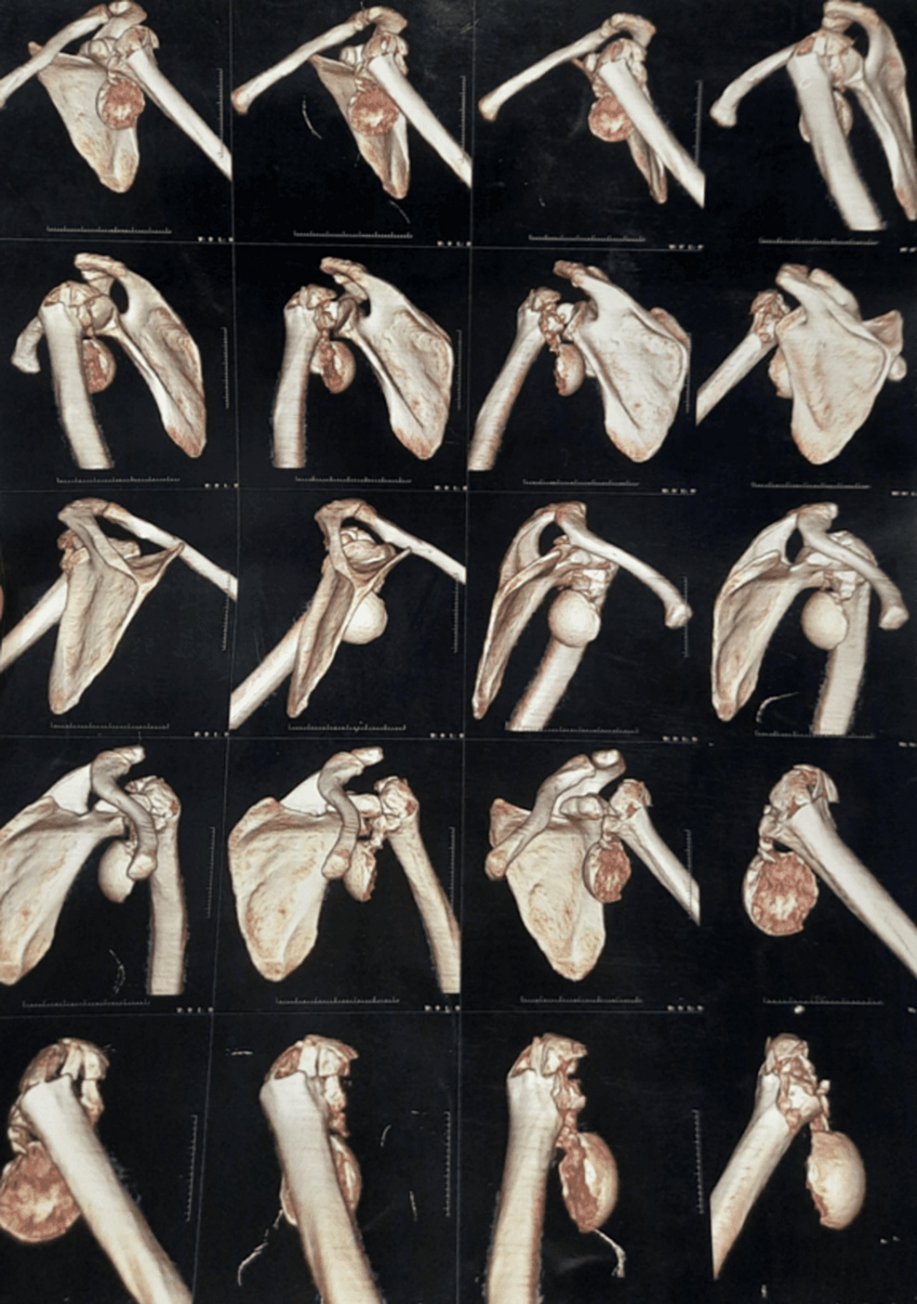
CT scan showing fracture and dislocation of left shoulder joint.

**Figure 3:**
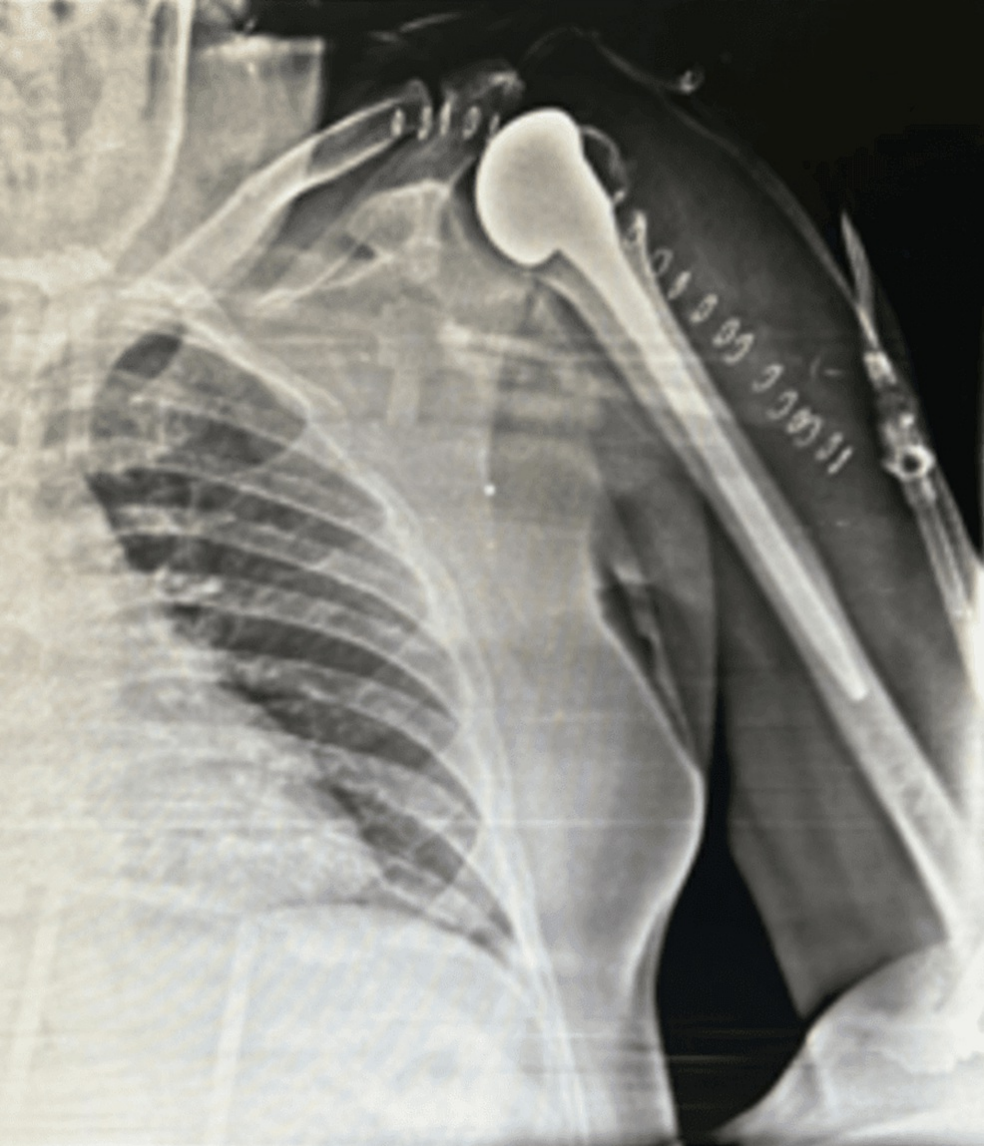
Post-operative X-ray of the left shoulder joint

A 58-year-old male with the dominance of the right side complained of severe pain and inability to perform left shoulder movements. These complaints commenced due to a road traffic accident on 13 April 2023. The patient had a fall over his left shoulder with a flexed elbow on a non-uniform road with stones from a two-wheeler. There was no history of loss of consciousness or any external bleeding. The patient was taken to a nearby hospital where a radiograph and CT scan of the left shoulder were obtained, which showed a displaced comminuted humeral head fracture with anteroinferior dislocation and fracture of the left shoulder (Figures 1, 2). Considering the severity of the comminuted fracture, left shoulder hemiarthroplasty was performed on 22 April 2023 (Figure 3). The patient was admitted for two weeks post-operation. He did not receive any physiotherapy treatment post-operation for one month due to a lack of awareness and knowledge about the same. He continued taking medications for pain relief. When the pain intensity increased, and he was unable to perform functional activities, the patient visited Dr. D. Y. Patil for physiotherapy OPD and physiotherapy rehabilitation treatment was started on 4 June 2023.

Clinical findings

The patient was assessed in a standing position with hands neutrally by the side of the body and head in a neutral position. On general examination, vitals were stable. In postural observation, left shoulder depression was observed. The scar was present over the anterior aspect of the left shoulder, which was 8cm long. When palpated, tenderness was absent over the shoulder region. On the numerical pain rating scale, the pain intensity was 9/10 while moving the shoulder joint in all planes and 2/10 while resting. The nature of the pain was intermittent, which aggravated while attempting to perform movements. On examination, the patient could not actively perform a specific shoulder range of motion. Initially, the patient could perform shoulder flexion, abduction, and internal rotation in a gravity-eliminating plane and the patient presented with a significant reduction in scapular mobility (Table 1). Pre-treatment strength assessment is mentioned in Table 2. On assessing the function based on the shoulder pain and disability index (SPADI), the score obtained was 86/130. The end feel of shoulder movements was empty due to pain. In sensory examination, superficial sensations, deep sensations, and cortical sensations were intact. No positive findings were captured in the gait except reduced arm swing on observation and balance assessment was normal.

**Table 1:** Pre-treatment ROM assessment ROM: range of motion

Joint	Movement	Active Left	Passive Left
Shoulder	Flexion	0-10°	0-120°
	Extension	0-10°	0-35°
	Abduction	0-5°	0-90°
	Adduction	5-0°	90-0°
Elbow	Flexion	0-140°	0-140°
	Extension	140-0°	140-0°

**Table 2:** Manual muscle testing for the left upper limb

Joint	Muscles	Grade
Shoulder	Flexors	1/5
	Extensors	1/5
	Abductors	1/5
Elbow	Flexors	4/5
	Extensors	4/5
Wrist	Flexors	4/5
	Extensors	4/5

Timeline

On 13 April 2023, the patient had a fall from a two-wheeler over the patient's left shoulder on a non-uniform road, from where he was taken to a nearby hospital where investigations were performed. The patient was operated on with left shoulder hemiarthroplasty on 22 April 2023. Post-operation, the patient was on pain medications, but no significant improvement was seen. So on 4 June 2023, he visited Dr. D. Y. Patil, physiotherapy outpatient department, and physiotherapy rehabilitation was started on the same day.

Diagnostic assessment

The day after the accident, an X-ray and CT scan were obtained in which a radiograph of the left shoulder revealed a displaced comminuted humeral head fracture with anterior dislocation of the left shoulder (Figure 1). CT scan revealed multiple small fragments in the adjacent soft tissue around the left shoulder. Also, glenohumeral joint effusion was noted which can be resolved by active physical therapy exercises helping them to drain in the lymph nodes nearby. The displacement of the humeral head was antero-inferiorly (Figure 2). The reports of Hb, WBC, RBC, and blood sugar levels were regular. As the fracture was highly impacted, the decision to operate it with shoulder hemiarthroplasty was taken into consideration. The post-operative radiograph showed a cemented unipolar implant, including a head resembling the humeral head placed in the glenoid cavity and steam reaching up to the upper 1/3rd of the shaft, indicating hemiarthroplasty (Figure 3). The patient was diagnosed as a postoperative case of left shoulder hemiarthroplasty.

Physiotherapy intervention

Physiotherapy intervention was built which consisted of short-term goals and long-term goals (Table 3). Short-term goals comprised the education of the patient regarding the condition and engaging the patient in all aspects of interventions. Prevention of muscle stiffness and post-operative complications and encompassing pain reduction techniques followed by re-education of muscle by electrical stimulation and increasing muscle strength was established. Eccentric strengthening helps in increasing strength when muscle fibres are lengthened. Implementation of a range of motion exercises in available range in gravity eliminating plane and further progressing to against gravity, along with wand exercises. The use of a shoulder wheel and finger ladder was also involved. Activation of muscle and stretching was incorporated. Long-term exercises were focused on maintaining short-term goals along with improving cardiopulmonary resilience and improving coordination through various exercises and helping the patient in returning back to normal recreational activities.

**Table 3:** Physiotherapy management Short-term goals: the duration was four weeks Long-term goals: the duration was five to six weeks

Short-Term Goals	Interventions	Dosage	Rationale
1) Patient education	Educate the patient about the importance of exercise for improving the quality of life.	At the beginning of treatment on Day 1	To make patient actively involved in the rehabilitation
2) Pain reduction	Hot pack	Before proceeding the exercises 10 min over the anterior aspect of the shoulder	Reduce pain and promote healing and make soft tissue more pliable.
3) Muscle activation	A) Electrical muscle stimulation over middle fibres of deltoid. B) Serratus anterior muscle activation. C) Rotator cuff muscle activation	A) Surged faradic current 30 contractions × 3 repetitions. B) & C) 10 repetitions 4 times in a day	To improve the contractility of the muscle and improve the strength of the musculature
4) Increase muscle strength	A) Eccentric muscle strengthening for shoulder flexors and abductors. B) exercise with resistance band.	10 repetitions 4 times in a day	To improve and maintain muscle strength
5) Improve range of motion	A) Shoulder ROM exercises in gravity eliminating plane in available range and further progressing to against gravity. B) wand exercises. C) Shoulder wheel. D) Finger ladder	15 repetitions 4 times in a day	To implement the required range of activities
6) Improve flexibility	Gentle stretching manoeuver to anterior muscle pectoralis major	3 sets of 30 seconds hold twice a day	Enhance flexibility thereby improving range
7) Maintain posture	A) Shoulder shrugs, B) scapular sets	15 repetitions 4 times in a day	To avoid depression of shoulder on one side
Long-Term Goals			
1) Improve neuromuscular control and muscle endurance	Stabilization exercises	Twice a day	To improve muscle endurance and control

Follow-up and outcome are mentioned in Table 4 and Table 5.

**Table 4:** Pre- and post-treatment range of motion comparison

Joint	Movement	Pre-treatment Active Left	Post-treatment Active Left
Shoulder	Flexion	0-10°	0-40°
	Extension	0-10°	0-35°
	Abduction	0-5°	0-25°
	Adduction	5-0°	25-0°
Elbow	Flexion	0-140°	0-140°
	Extension	140-0°	140-0°

**Table 5:** Pre- and post-treatment of MMT comparison MMT: Methadone maintenance treatment

Joint	Muscles	Pre-treatment MMT	Post-treatment MMT
Shoulder	Flexors	1/5	3+/5
	Extensors	1/5	3+/5
	Abductors	1/5	3/5
Elbow	Flexors	4/5	5/5
	Extensors	4/5	5/5
Wrist	Flexors	4/5	5/5
	Extensors	4/5	5/5

## Discussion

Shoulder hemiarthroplasty is a surgical procedure in which the shoulder joint is removed and substituted or replaced with artificial implants. The advantage is restoration of the functional mobility of the shoulder joint, in association with reducing pain and other complaints. Regaining the range of motion strength and following the restoration of functional activity of the shoulder joint are essential for patients to obtain a decent and good outcome post-surgery [6]. In the present case, the patient presented with left shoulder hemiarthroplasty with complaints of restricted range of motion and inability to perform functional activities of the left shoulder joint. Physiotherapy rehabilitation here can play an essential role in resolving patients' problem list as the root cause for impaired and restricted shoulder ranges. Physiotherapy can be effective in reducing pain, activating the muscles, and improving strength, thereby contributing to good functional status and good quality of life. In the given Table 1 are the ranges recorded at the beginning of the assessment where the surgery patient could still not perform initial ranges of shoulder flexion, abduction, extension, and internal-external rotation. Similarly, with altered ranges, the patient also lacked the strength in muscles to activate respective action at the joint level, which again is a contributing cause to loss of ranges. Table 3 states the strength of muscles on the first day of assessment. A tailored protocol with sufficient dosage and progression was introduced which showed a gradual progression in the range of motion and strength of the individual.

The given case study varies from other cases; there was an introduction of muscle stimulation using faradic current to see the effectiveness of pain reduction and muscle activation, which resulted in the improvement of ranges and muscle strength (Table 2 and Table 4). In addition to using electrical stimulation, there was the contribution of exercises that focused on muscle activation in the initial stages, which combined gave an improved functional response. The entire treatment protocol played a crucial role in reducing pain and boosting muscle strength, thereby increasing and enhancing ranges and functional self-dependence, and quality of life in patients with total shoulder hemiarthroplasty [7-10].

## Conclusions

We conclude that the application of physical therapy rehabilitation in patients with shoulder hemiarthroplasty helps in improving the well-being of the patient. The case study differs in the occurrence of severe comminuted fracture of the head of the humerus along with dislocation which rarely occurs in road traffic accidents. Thus implementation of physical therapy plays a vital role in getting back a patient to normal daily activities by minimizing pain and enhancing functional range of motion and gaining required strength.
